# Predictive Factors of 30-day Adverse Events in Acute Heart Failure after Discharge from Emergency Department; a Historical Cohort Study

**DOI:** 10.22037/aaem.v9i1.1271

**Published:** 2021-09-01

**Authors:** Siriwimon Tantarattanapong, Keerati Keeratipongpun

**Affiliations:** 1 Department of Emergency Medicine, Songklanagarind Hospital, Faculty of Medicine, Prince of Songkla University, Hat Yai, Songkhla, Thailand

**Keywords:** Heart failure, Patient discharge, Emergency service, hospital, Patient readmission, Patient admission

## Abstract

**Introduction:**

The rates of unscheduled emergency department (ED) visits and readmissions after discharge from the ED in acute heart failure (AHF) patients are high. This study aimed to identify the predictive factors of 30-day adverse events after discharge from the ED.

**Methods:**

A retrospective study was conducted from 2017 to 2019 in patients diagnosed with AHF and discharged from the ED at a tertiary university hospital. Thirty-day adverse events were defined as (i) unscheduled revisit to the ED with AHF, (ii) hospital admission from AHF, and, (iii) death after discharge from the ED. The predictive factors of 30-day adverse events were examined using multivariate analyses by logistic regression.

**Results:**

421 patients with the median age of 73 (IQR: 63-81) years were studied (52.3% male). 81 (19.2%) patients had 30-day adverse events. Significant predictive factors of 30-day adverse events consisted of underlying valvular heart disease (OR = 2.46; 95%CI: 1.27-4.78; p = 0.008), chronic obstructive pulmonary disease (COPD) (OR = 0.08; 95%CI: 0.01-0.64; p=0.001), malignancy (OR=3.63; 95%CI: 1.17-11.24; p = 0.031), New York Heart Association functional class III (OR = 4.88; 95%CI: 0.93-25.59) and IV (OR = 7.23; 95% CI: 1.37-38.08) at the ED (p = 0.035), and serum sodium <135 mmol/L (OR = 2.20; 95%CI: 1.17-4.14; p = 0.014). Precipitating factors were anemia (OR = 2.42; 95%CI: 1.16-5.02; p = 0.021), progressive valvular heart disease (OR = 3.52; 95%CI: 1.35-7.85; p = 0.009), acute kidney injury (OR = 6.98; 95%CI: 2.32-20.96; p < 0.001), time to diuretic administration >60 minutes after ED arrival (OR = 3.89; 95%CI: 2.16-7.00; p < 0.001), and no discharge advice for follow-up (OR = 2.30; 95%CI: 1.10-4.77; p = 0.028).

**Conclusion:**

AHF patients who had good response to intravenous diuretics and were discharged from the ED were at high risk for 30-day adverse events. Ten factors predicted 30-day adverse events after discharge from the ED.

## 1. Introduction:

Acute heart failure (AHF) is defined as a rapid onset of clinical syndromes of heart failure such as dyspnea, fatigue, pulmonary congestion, and peripheral edema, which can be new onset (de novo) or due to the worsening of preexisting heart failure (1). The incidence of AHF is approximately 1 million cases per year in the United States of America (2). The patients present to emergency departments (EDs) and emergency physicians (EP) evaluate, diagnose, and treat AHF patients. The decision making for disposition is crucial and a challenging issue for the EP. If the EP makes a decision that results in an inappropriate disposition, the risks of morbidity and mortality will increase (3, 4). About 16% to 36% of AHF patients can be discharged from the ED after a brief period of observation (3-6). The rate of unscheduled ED visits was reported to be 26% and readmission rate was 15% in 30 days (4). The 7-day and 30-day mortality rates in AHF patients after discharge from the ED were reported to be 2% and 3.3%, respectively (3, 5). From a previous study, the factors that influenced an unscheduled ED visit and readmission of AHF patients after discharge from the ED included underlying ischemic heart disease, pulmonary disease, valvular heart disease, anemia, malignancy, glomerular filtration rate (GFR) <60 mL/min/1.73 m^2^, and no administration of an intravenous diuretic agent (4-6). The factors that predicted AHF mortality were underlying ischemic heart disease, pulmonary disease, valvular heart disease, malignancy, and elevated potassium and troponin-T levels, whereas consumption of angiotensin-converting enzyme inhibitor, angiotensin II receptor blocker, beta-blocker, and spironolactone were associated with lower mortality rates (4-6).

Consideration for discharge from the ED depends on the clinical condition, good response to initial therapy, and no de novo AHF (7). However, the risk factors that influence an unscheduled ED visit, readmission, or mortality are not considered in the discharge criteria. The aim of this study was to identify the predictive factors of 30-day adverse events in AHF patients after discharge from the ED to help the EPs make appropriate dispositions.

## 2. Methods

***2.1. Study design and setting***

A retrospective cohort study was conducted at the ED of Songklanagarind Hospital, which is a tertiary university hospital in southern Thailand. The data were collected from February 2017 to June 2019. Ethics approval was obtained from the Institutional Ethics Committee Board of the Faculty of Medicine at Prince of Songkla University (Ethics code: REC.62-167-20-4).

***2.2. Participants***

The inclusion criteria were patients aged ≥15 years, diagnosed with AHF, and discharged home from the ED based on the decision of the EP and internists. Patients admitted or referred to other hospitals were not included in this study. The exclusion criteria were (i) end-stage renal disease with hemodialysis or peritoneal dialysis, (ii) pregnancy, (iii) incomplete discharge criteria, and (iv) lost to follow-up.

***Procedure***

When the patients presented to the ED, the EP evaluated the history, performed physical examination, and carried out investigations to diagnose AHF according to the Framingham criteria and echocardiography. The Framingham criteria consist of (i) acute pulmonary edema, (ii) cardiomegaly, (iii) hepatojugular reflex, (iv) neck vein distension, (v) paroxysmal nocturnal dyspnea or orthopnea, (vi) rales, and (vii) third heart sound gallop. The minor criteria consist of (i) ankle edema, (ii) dyspnea on exertion, (iii) hepatomegaly, (iv) nocturnal cough, (v) pleural effusion, and (vi) tachycardia (>120 beats/minute). AHF was diagnosed when two major criteria or one major and two minor criteria were met (8). After the patients were diagnosed, the EP looked for and worked up the precipitating causes and management of AHF using intravenous diuretics. The response was observed to fulfill the discharge criteria. Criteria for discharge consisted of (i) patient-reported subjective improvement, (ii) resting heart rate <100 bpm, (iii) no hypotension, (iv) adequate urine output, (v) oxygen saturation >95% in room air, and (vi) no de novo heart failure (7). Adequate urine output was defined as urine output >100 mL/h after intravenous diuretic administration (9). 

***2.3. Data gathering***

The data collected from the medical records included patient baseline characteristics, history taking, physical examination, precipitating factors, diagnosis, investigations, treatments, and 30-day adverse events. Thirty-day adverse events were defined as (i) unscheduled revisit to the ED with AHF within 30 days, (ii) hospital admission due to AHF within 30 days after discharge from the ED, and (iii) death within 30 days after discharge from the ED.

Time to diuretic administration was considered as the interval from the time the patient entered the ED until the patient received the first dose of an intravenous diuretic agent. 

***2.4. Outcome measurements***

The primary outcome was to identify the predictive factors of 30-day adverse events in AHF patients who were discharged home from the ED. The secondary outcome was to identify the incidence of 30-day adverse events.

***2.5. Statistical analysis***

The sample size was calculated using the n4Studies based on a study by Miró et al. (4). The statistical analysis was conducted using R software version 3.6.1. Continuous variables were analyzed and are reported as median and interquartile range (IQR), while discrete variables are reported as percentage. All data were based on non-parametric frequency distributions. The univariate model analyzed the baseline characteristics, clinical presentations, investigations, and treatments. The data were compared between subjects with and without 30-day adverse events after discharge from the ED. Continuous variables were compared using the Mann-Whitney U-test. Categorical variables were compared using the χ^2^ test or Fisher’s exact test as indicated. Significant predictive factors associated with 30-day adverse events in AHF (p < 0.2) identified during the univariate analysis were introduced into a logistic regression model with backward stepwise selection. First-order interaction terms with combinations of all independent predictors were introduced into the multivariate model one at a time. Generally, interaction terms were considered with statistical significance set at p < 0.05 and no significant interaction between the included variables in the final logistic regression models. Modeling results are shown as odds ratio (OR) with 95% confidence interval (CI). A two-tailed p < 0.05 was selected as the level of statistical significance.

## 3. Results

***3.1. Baseline characteristics***

From a total of 614 AHF patients, 421 met the inclusion criteria of this study. The median age was 73 (IQR 63-81) years (52.3% male). The percentages of patients with underlying valvular heart disease (50.6% vs. 27.4%; p < 0.001) and malignancy (11.1% vs. 4.1%; p = 0.025) were significantly higher in the 30-day adverse event group. None of the medications used by the patients were statistically different between the two groups (Table 1). 

In patients with adverse events, both median systolic and diastolic blood pressures were lower than the no adverse event group (Table 2). The mean serum sodium level in the 30-day adverse event group was also lower. The statistically significant precipitating factors that led to 30-day adverse events were anemia, non-compliance with dietary restrictions, acute kidney injury, and progressive valvular heart disease. 

The median times to diuretic administration in the no 30-day adverse event group and 30-day adverse event group were 53 minutes and 70 minutes, respectively (p = 0.040). The median total doses of diuretic agents were higher in the 30-day adverse event group (Table 3).

***3.2. Outcomes***

**Figure 1:**
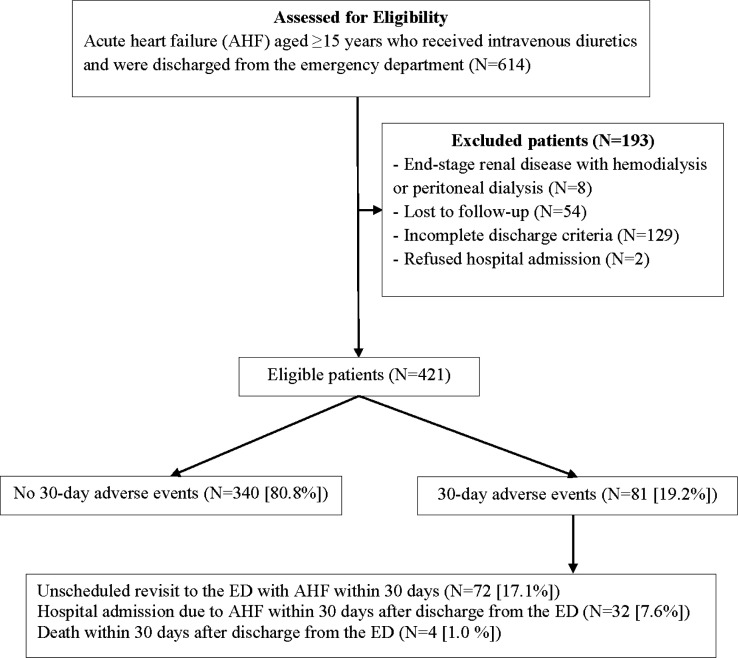
Study inclusion flow diagram. ED: emergency department

The incidence rate of 30-day adverse events after discharge from the ED was 19.2% (81/421). The rates of unscheduled revisit, hospital admission, and mortality within 30 days after discharge from the ED were 17.1%, 7.6%, and 1.0%, respectively (Figure 1). Based on multivariate analysis, the factors that increased the odds of 30-day adverse events included underlying valvular heart disease, malignancy, New York Heart Association (NYHA) functional class III and IV at the ED, and sodium level <135 mmol/L. The precipitating factors were anemia, progressive valvular heart disease, acute kidney injury, door-to-diuretic time >60 minutes, and no advice for follow-up (Table 4). However, COPD was a predictive factor that decreased 30-day adverse events.

## 4. Discussion

The overall incidence of adverse events in AHF patients discharged from the ED was 19.2%, which consisted of unscheduled revisits at the ED within 30 days (17.1%), hospital admission within 30 days (7.6%), and death within 30 days (1.0%). The incidences of admission to the hospital within 30 days and death within 30 days were close to previous studies at 15.7% and 1.7-4.0%, respectively (4, 5). 

This study revealed the predictive factors associated with 30-day adverse events after discharge from the ED, which included underlying valvular heart disease, malignancy, COPD, NYHA functional class III and IV at the ED, and serum sodium level. In addition, the precipitating factors were identified to be anemia, progressive valvular heart disease, acute kidney injury, time to diuretic administration, and no advice for follow-up. 

The predictive factors that increased 30-day adverse events after discharge home from the ED in this study consisted of underlying valvular heart disease (adjusted OR [adj. OR] = 2.46; 95%CI: 1.27-4.78) (p = 0.008), malignancy (adj. OR = 3.63; 95%CI: 1.17-11.24) (p = 0.031), and NYHA functional class III and IV at the ED (adj. OR = 4.88; 95%CI: 0.93-25.59 and adj. OR = 7.23; 95%CI: 1.37-38.08, respectively) (p = 0.035), which were quite similar to previous studies (4, 6). In this study, COPD was a predictive factor that decreased 30-day adverse events (adj. OR = 0.08; 95%CI: 0.01-0.64) (p = 0.001), which was contrary to other studies (3, 4). This inconsistency may have resulted due to different prevalence rates of COPD between the studies. The prevalence of COPD in this study was 5.9%, whereas the prevalence rates of COPD in two previous studies were 21.8% and 25.5% (3, 4). The lower prevalence of COPD in this study was possibly due to different inclusion criteria. This study included only AHF patients who were discharged directly from the ED, whereas the cited studies included AHF patients discharged from the ED and AHF patients admitted to the hospital. Patients admitted to the hospital generally have an underlying disease such as COPD. Therefore, this study reported fewer patients with COPD.

Lower serum sodium level was an associated factor for increased mortality in AHF patients (10). A serum sodium level <135 mmol/L was a predictive factor of adverse events in this study (adj. OR = 2.20; 95%CI: 1.17-4.14) (p = 0.014).

The time to diuretic administration influenced hospital mortality rate. A study by Matsue et al. and Maisel reported that ED arrivals who received an intravenous diuretic agent within 60 minutes in AHF patients had a lower hospital mortality rate (11, 12). The results of this study showed that a door-to-diuretic time >60 minutes predicted 30-day adverse events (adj. OR = 3.89; 95%CI: 2.16-7.00) (p < 0.001). Therefore, the EP should give early administration of intravenous diuretic agents within 60 minutes in AHF patients to decrease the occurrence of adverse events.

Significant precipitating factors to predict 30-day adverse events in this study were anemia (adj. OR = 2.42; 95%CI: 1.16-5.02) (p = 0.021), progressive valvular heart disease (adj. OR = 3.52; 95%CI: 1.35-7.85) (p = 0.009), and acute kidney injury (adj. OR = 6.98; 95%CI: 2.32-20.96) (p < 0.001). The EP should consider these factors in addition to the response to initial treatment prior to discharge. In accordance with the 2015 recommendations of European Society of Cardiology, the EP should identify low-risk features, absence of any known high-risk features, and a good response to the initial treatment for a safe discharge (7). Examples of high-risk features are significantly elevated natriuretic peptide levels, low blood pressure, worsening renal failure, hyponatremia, and positive troponin.

In AHF patients who have a good response to initial therapy and are considered for discharge home, the EP must give discharge instructions to present for follow-up within 72 hours (7). A study by VanSuch demonstrated that when all discharge instructions were not given, the AHF patients had an increased risk for readmission (13). In this study, no advice for follow-up was a predictive factor of 30-day adverse events (adj. OR = 2.30; 95%CI: 1.10-4.77) (p = 0.028). Ideally the patients should present for follow-up within 72 hours after discharge, but this depends on the hospital context. The patients should follow the instructions and return to the ED.

The EP should make the decision for a safe discharge based on good response to initial treatment, low-risk features, and an organized system for follow-up. However, low-risk and high-risk features to predict 30-day adverse events should be researched further in the future.

**Table 1:** Comparing the baseline characteristics between acute heart failure patients with and without 30-day adverse outcome

**Variables**	**30-day adverse event**		**p value**
	**With ** **(** **n** **=** **81** **)**	**Without** ** (** **n** **=** **340** **)**	
**Age (years)**			
Median (IQR)	73 (62,79)	73 (62,81)	0.305
**Sex**			
Male	36 (44.4)	184 (54.1)	0.149
Female	45 (55.6)	156 (45.9)	
**Baseline NYHA functional class**			
I	3 (3.7)	37 (10.9)	0.227
II	35 (43.2)	132 (38.8)	
III	26 (32.1)	89 (26.2)	
IV	2 (2.5)	17 (5.0)	
No record	15 (18.5)	65 (19.1)	
**Comorbidity**			
Hypertension	49 (60.5)	230 (67.6)	0.274
Diabetes mellitus	32 (39.5)	119 (35.0)	0.528
Hyperlipidemia	34 (42.0)	138 (40.6)	0.918
Ischemic heart disease	46 (56.8)	184 (54.1)	0.757
Valvular heart disease	41 (50.6)	93 (27.4)	<0.001
Atrial fibrillation	20 (24.7)	71 (20.9)	0.550
Chronic kidney disease	29 (35.8)	90 (26.5)	0.124
Cerebrovascular disease	12 (14.8)	37 (10.9)	0.424
COPD	1 (1.2)	24 (7.1)	0.063
Malignancy	9 (11.1)	14 (4.1)	0.025
Peripheral arterial disease	2 (2.5)	9 (2.6)	1.000
**Medication used**			
Diuretics	70 (86.4)	272 (80.0)	0.241
ACEI	11 (13.6)	82 (24.1)	0.057
ARB	5 (6.2)	49 (14.4)	0.071
Beta-blocker	47 (58.0)	213 (62.6)	0.521
Nitrate	20 (24.7)	66 (19.4)	0.365
Digoxin	10 (12.3)	32 (9.4)	0.558

Data are presented as n (%) or median and interquartile range (IQR). NYHA: New York Heart Association; COPD: chronic obstructive pulmonary disease; ACEI: angiotensin-converting enzyme inhibitors; ARB: angiotensin receptor blockers.

**Table 2:** Comparing the clinical presentations and laboratory findings between acute heart failure patients with and without 30-day adverse outcome

**Variables**	**30-day adverse event**		**p value**	
	**With ** **(** **n** **=** **81** **)**	**Without** ** (** **n** **=** **340** **)**		
**NYHA functional class at the ED**					
I	0 (0)	0 (0)		0.050		
II	2 (2.5)	31 (9.1)				
III	29 (35.8)	138 (40.6)				
IV	36 (44.4)	105 (30.9)				
No record	14 (17.3)	66 (19.4)				
**Initial vital signs**					
SBP (mmHg)	123.0 (110.0,143.0)	138.0(120.0,154.2)		<0.001		
DBP (mmHg)	70.0 (61.0,79.0)	76.5 (65.0,87.0)		0.001		
PR (bpm)	80.0 (70.0,96.0)	82.0 (70.0,96.0)		0.967		
RR (breaths/minute)	28.0 (24.0,32.0)	28.0 (24.0,32.0)		0.499		
SpO_2 _(%)	97.0 (95.0,99.0)	97.0 (95.0,99.0)		0.648		
**Laboratory findings**					
GFR (mL/min/1.73 m^2^)	48.5 (32.0,67.0)	57.5 (37.2,75.8)		0.097		
Sodium (mmol/L)	136.1 (133.0,140.8)	138.9 (135.6,141.5)		<0.001		
Potassium (mmol/L)	3.9 (3.5,4.3)	4.0 (3.6,4.3)		0.447		
Troponin-T (ng/mL)	44.8 (29.8,70.3)	34.6 (19.4,62.4)		0.032		
Pro-BNP (pg/mL)	8,041.0 (3,349.0-18,394.0)	4,805.0 (2,181.5-9,590.0)		0.110		
**Precipitating factors**					
Infection	14 (17.3)	46 (13.5)		0.489		
Non-STEACS	5 (6.2)	10 (2.9)		0.179		
Anemia	20 (24.7)	48 (14.1)		0.031		
Poor drug compliance	16 (19.8)	92 (27.1)		0.226		
Non-compliance with DR	16 (19.8)	115 (33.8)		0.020		
Arrhythmia	3 (3.7)	15 (4.4)		1.000		
Hypertension	4 (4.9)	26 (7.6)		0.541		
Acute kidney injury	10 (12.3)	11 (3.2)		0.002		
Progressive VHD	19 (23.5)	24 (7.1)		<0.001		

Data are presented as number (%) or median and interquartile range (IQR). NYHA: New York Heart Association; ED: emergency department; SBP: systolic blood pressure; DBP: diastolic blood pressure; PR: pulse rate; RR: respiratory rate; SpO_2_: oxygen saturation; DR: dietary restrictions; VHD: valvular heart disease; GFR: glomerular filtration rate; Pro-BNP: N-terminal pro B-type natriuretic peptide; Non-STEACS: Non-ST elevation acute coronary syndromes.

**Table 3:** Comparing the emergency department management and disposition characteristics between acute heart failure patients with and without 30-day adverse outcome

**Variables**	**30-day adverse event **		**p value**
	**With ** **(** **n** **=** **81** **)**	**Without** ** (** **n** **=** **340** **)**	
**Medication**			
Time to diuretic (minutes)	70.0 (40.0,100.0)	53 (36.0,90.0)	0.040
Initial dose of IV diuretics (mg)	40.0 (40.0,80.0)	40.0 (40.0,80.0)	0.170
Total dose of IV diuretics (mg)	120.0 (40.0,240.0)	80.0 (40.0,130.0)	0.010
**Consult with internist**			
Yes	23 (28.4)	75 (22.1)	0.286
**Discharge instruction items**			
Lifestyle modification	22 (27.2)	88 (25.9)	0.925
Drug compliance	17 (21.0)	54 (15.9)	0.348
Warning signs and symptoms	40 (49.4)	134 (39.4)	0.130
Advice for follow-up	61 (75.3)	295 (86.8)	0.017
**ED length of stay (hours)**			
Median (IQR)	4.9 (3.5,6.1)	4.5 (3.3,5.6)	0.166

Data are presented as number (%) or median and interquartile range (IQR). IV: intravenous; ED: emergency department.

**Table 4:** Predictive factors of 30-day adverse events of patients with acute heart failure who were discharged from the emergency department based on the results of multivariate logistic regression analysis

**Variables**	**Crude OR **	**Adjusted OR **	**p value**
**Comorbidity **			
Valvular heart disease	2.72 (1.66-4.47)	2.46 (1.27-4.78)	0.008
COPD	0.16 (0.02-1.23)	0.08 (0.01-0.64)	0.001
Malignancy	2.91 (1.21-6.99)	3.63 (1.17-11.24)	0.031
**NYHA functional class at ED**			
III	3.26 (0.74-14.38)	4.88 (0.93-25.59)	0.035
IV	5.31 (1.21-23.33)	7.23 (1.37-38.08)	
**Precipitating factors**			
Anemia	1.99 (1.11-3.60)	2.42 (1.16-5.02)	0.021
Progressive valvular heart disease	4.03 (2.08-7.81)	3.52 (1.35-7.85)	0.009
Acute kidney injury	4.21 (1.72-10.3)	6.98 (2.32-20.96)	<0.001
**Door-to-diuretic time >60 min**	2.67 (1.62-4.4)	3.89 (2.16-7.00)	<0.001
**Sodium <135 mmol/L**	2.07 (1.22-3.53)	2.20 (1.17-4.14)	0.014
**No advice for follow-up**	2.15 (1.19-3.89)	2.30 (1.10-4.77)	0.028

Data are presented with 95% confidence interval; OR: odds ratio; COPD: chronic obstructive pulmonary disease; NYHA: New York Heart Association; ED: emergency department.

## 5. Limitations

This study was a single-center retrospective study. Therefore, missing data on the follow-up of patients affected the mortality rate due to the unknown status of some patients. 

## 6. Conclusion

It seems that AHF patients who have good response to intravenous diuretics and are discharged from the ED are at high risk for 30-day adverse events. The significant factors to predict an increased risk of 30-day adverse events after discharge from the ED consisted of underlying valvular heart disease, malignancy, NYHA functional class III and IV at the ED, and serum sodium level <135 mmol/L. The precipitating factors were anemia, progressive valvular heart disease, acute kidney injury, time to diuretic administration >60 minutes after ED arrival, and no advice for follow-up as part of discharge instructions. On the other hand, COPD was a low risk factor of 30-day adverse events in this study.

## 7. Declarations

### 7.1. Acknowledgments

The authors thank Kingkarn Waiyanak for search and retrieval of articles, Glenn K. Shingledecker for his help in editing the manuscript, and the Faculty of Medicine for funding this research.

### 7.2. Author contributions

Keerati Keeratipongpun and Siriwimon Tantarattanapong performed the literature research, study design, data collection, data analysis, data interpretation, and writing the manuscript. Both authors contributed to data analysis, drafting, and the critical revisions of the paper and agree to be accountable for all aspects of the work.

### 7.3. Funding and supports

The Faculty of Medicine, Prince of Songkla University funded this research.

### 7.4. Conflict of interest

The authors declare they have no conflict of interest.
